# Interferon γ induced compositional changes in human bone marrow derived mesenchymal stem/stromal cells

**DOI:** 10.1186/s12014-017-9161-1

**Published:** 2017-07-06

**Authors:** Qingdong Guan, Peyman Ezzati, Victor Spicer, Oleg Krokhin, Donna Wall, John A. Wilkins

**Affiliations:** 1 Manitoba Centre for Advanced Cell and Tissue Therapy, Department of Pediatrics and Child Health, University of Manitoba, Winnipeg, MB Canada; 2 Cellular Therapy Laboratory, CancerCare Manitoba, Winnipeg, MB Canada; 3 Manitoba Centre for Proteomics and Systems Biology, Section of Biomedical Proteomics, Department of Internal Medicine, Rady Faculty of Health Sciences, University of Manitoba and Health Sciences Centre, 799 John Buhler Research Centre, 715 McDermot Ave, Winnipeg, MB R3E 3P4 Canada; 4 Division of Haematology/Oncology, Department of Paediatrics, The Hospital for Sick Children, University of Toronto, Toronto, ON Canada

**Keywords:** Proteomics, Human, 2D LC mass spectrometry, Interferon γ, Mesenchymal stem/stromal cells (MSC), Quantitative proteome profiling, Licensing, Membrane

## Abstract

**Background:**

Mesenchymal stem/stromal cells (MSC) display a range of immunoregulatory properties which can be enhanced by the exposure to cytokines such interferon γ (IFN-γ). However the compositional changes associated with the ‘licensing’ of these cells have not been clearly defined. The present study was undertaken to provide a detailed comparative proteomic analysis of the compositional changes that occur in human bone marrow derived MSC following 20 h treatment with IFN-γ.

**Methods:**

2D LC MSMS analysis of control and IFN-γ treated cells from 5 different healthy donors provided confident identification of more than 8400 proteins.

**Results:**

In total 210 proteins were shown to be significantly altered in their expression levels (≥|2SD|) following IFN-γ treatment. The changes for several of these proteins were confirmed by flow cytometry. STRING analysis determined that approximately 30% of the altered proteins physically interacted in described interferon mediated processes. Comparison of the list of proteins that were identified as changed in the proteomic analysis with data for the same proteins in the Interferome DB indicated that ~35% of these proteins have not been reported to be IFN-γ responsive in a range of cell types.

**Conclusions:**

This data provides an in depth analysis of the proteome of basal and IFN-γ treated human mesenchymal stem cells and it identifies a number of novel proteins that may contribute to the immunoregulatory capacity if IFN-γ licensed cells.

**Electronic supplementary material:**

The online version of this article (doi:10.1186/s12014-017-9161-1) contains supplementary material, which is available to authorized users.

## Background

Mesenchymal stem/stromal cells (MSC) are multipotent stromal cells derived from all mammalian supportive stromal tissue compartments containing distinct pools of endogenous progenitor cells. MSC possess the potential for self-renewal and multi-lineage differentiation. The demonstration that MSC can also display potent immunoregulatory activities has led to marked interest in their potential use in the treatment of autoimmunity and transplant rejection [1–4]. The underlying processes include both contact dependent (e.g. PDL-1) and soluble effector mediated processes (e.g. IDO-1) [5, 6]. Additionally, MSC can regulate innate and adaptive immune responses through the release of soluble mediators (e.g. IL-10, TGFβ), the induction of regulatory T cells (Treg) and the suppression effector CD4+ and CD8+ T cells [7–11]. The fact that this activity may be enhanced by treatment of MSC with cytokines such as TNF α, IFN-γ, IL-17, IL-1α or IL-1β, has resulted in several protocols for possible ‘licensing’ of MSC [12–15]. Although a number of pathways have been implicated in this process, the underlying mechanisms responsible for the immunoregulatory activity have not been fully elucidated. These observations highlight the need for an understanding of the responses of MSC to treatment with the cytokines.

To date there have been a limited number of detailed analysis of the proteomes of MSC. In many of these studies, the focus has been largely on the comparative analysis of cells from different tissue sources, and/or multi-tissue differentiation ability, tissue repair and self-renewal [16, 17] or more recently relating to the secretomes of various cells [18–20]. Significantly the majority of these studies have used fetal bovine serum (FBS) expanded MSC for proteomics analysis which will not be used in the manufacturing of clinical grade MSC. In fact there have been very few studies that have examined MSC and their responses to cytokine stimulation. Thus the basis for cytokine enhanced MSC immunoregulatory activity is not fully understood.

The aim of the present study was to determine the changes in protein composition that occur in GMP grade human platelet lysate expanded MSC subsequent to 20 h of treatment with interferon γ. The focus was specifically on those proteins that displayed consistent and significant alterations in their expression patterns following treatment. These proteins might ultimately be useful in identifying markers to assess the IFN-γ responses of treated cells or in defining the compositional changes mechanistically involved in the immunoregulatory features of the licensed cells.

## Methods

### Chemicals

All chemicals were sourced from Sigma Chemicals (St-Louis, MO), unless noted otherwise. HPLC-grade acetonitrile and de-ionized water were used for the preparation of eluents. Sequencing-grade modified trypsin (Promega, Madison, WI) and 15 mL Amicon centrifugal filter units (Merck Millipore, Ireland) was used for digestion. Siliconized 1.5 mL vials (BioPlas, San Rafael, CA) were used for all sample preparation and fractions handling steps.

### MSC expansion and licensing

Bone marrow aspirates were obtained from the posterior iliac crest of normal volunteers under institutional REB-approved study. The donors were 4 males and 1 female ranging in age from 8 to 41 years. Bone marrow-derived MSC cultures were established and maintained in a GMP compliant facility as described previously [21]. To license MSC, cells from the first or second passage were seeded into T175 flasks at 2000–3000 cells/cm^2^ in complete DMEM media (Lonza, USA) with 5% human platelet lysate (Mill Creek Life Sciences, Rochester, USA), 1% glutamax (Life technologies, California, USA) and gentamicin (5 µg/ml, Life Technologies); When the cell confluence reached 70–80%, human recombinant IFN-γ (eBioscience, California, USA) was added into the MSC culture media at 30 ng/mL for 20 h and then cells were released with tryple select (Life Technologies), washed with DPBS and analyzed [22].

### Phenotyping of MSC

The immunophenotype of expanded MSCs was characterized by flow cytometry as described previously (Minimal criteria for defining multipotent mesenchymal stromal cells [23]. Briefly, 0.5 × 10^6^ MSCs were washed in FACS buffer, and then incubated with Fc block for 10 min. This was followed by staining with FITC, APC or PE -labelled antibodies against surface molecules CD90, CD73, CD105, CD34, CD45, CD14, HLA-DR, CD19 and isotype control (eBioscience) respectively, for 20 min according to the manufacturer recommendations. After staining, cells were acquired and analyzed using flow cytometry (FACS Canto II, BD Biosciences, San Jose, USA) and FlowJo software (TreeStar, San Carlos, CA, USA).

Using the same methods, PE-Cy7, FITC, APC-Cy7 or Percp-Cy5.5 labeled antibodies against PD-L1, BST-2, ICAM-1 and VCAM-1 (eBioscience), were used to evaluate the expression of PD-L1, BST-2, ICAM-1 and VCAM-1 on the surface of MSC. To evaluate the expression of intracellular proteins IDO-1, MSC were fixed and permeabilized using eBioscience intracellular fixation/permeabilization buffer. Cells were then stained with PE-labeled anti-IDO-1 for 20 min. After staining, cells were acquired and analyzed using flow cytometry (FACS Canto II, BD Biosciences, San Jose, USA) and FlowJo software (TreeStar, San Carlos, CA, USA).

### MSC trilineage differentiation assay

MSC were induced to differentiate into adipogenic, osteogenic or chondrogenic lineages, with the use of the STEMPRO Adipogenesis, Osteogenesis, and Chondrogenesis Differentiation Kit (Thermo Fisher Scientific) as described previously [24]. Briefly, MSC were seeded into 6-well plates at 3000/cm^2^ in DMEM with 5% human platelet lysate. When MSC were reached 80% confluency, each well was replaced with adipogenic, osteogenic or chondrogenic differentiation media according to the manufacturer’s instruction for 14–21 days. The staining analysis was performed with the use of oil red O, Alizarin red S and Safranin O staining for adipocytes, osteocytes and chondrocytes, respectively.

### Processing of cells for MS analysis

Tryptic digests of MSC were prepared using the scaled up (15 ml filter units) FASP digestion procedure [25]. Protein amounts to be subjected to digestion were monitored using micro-BCA assay (Pierce, Rockford, IL). Resulting digest was acidified with TFA and purified by RP SPE. Approximately 100 μg of the digests (determined by NanoDrop 2000, ThermoFisher) was used for 2D LC–MS experiments [26].

### First dimension separation and fraction collection

Agilent 1100 series LC system with UV detector (214 nm) and 3 mm × 100 mm XTerra MS C18, 3.5 μm column (Waters, Ireland) was used for pH 10 separations. 0.66% acetonitrile gradient (0–40% acetonitrile) was delivered at 300 μL/min flow rate. Both eluents A (water) and B (1:9 water:acetonitrile) contained 20 mM ammonium formate and were prepared by 1:10 dilution of 200 mM ammonium solution with pH 10 adjusted by formic acid. Manual Reodyne injector (Bensheim, Germany) with 200 μL loop was used to deliver ~100 μg of digested sample onto the column. One-minute fractions were collected over the 7–55 min interval, concatenated into 21 fractions, lyophilized and re-suspended in buffer A (0.1% formic acid in water).

### HPLC–MS settings in the second dimension

A splitless nano-flow 2D LC Ultra system (Eksigent, Dublin, CA) was used to deliver water/acetonitrile gradient at 500 nL/min flow rate through a 100 μm × 200 mm analytical column packed with 3 μm Luna C18(2) (Phenomenex, Torrance, CA) at room temperature. Sample injection (~1 μg of peptides from each fraction in 10 μL of buffer A) via a 300 μm × 5 mm PepMap100 trap-column (ThermoFisher) was used in all experiments. The gradient program included following steps: linear increase from 0.5 to 30% of buffer B (acetonitrile) in 78 min, 5 min columns wash with 90% B and 8 min system equilibration using starting conditions of 0.5% B. Both eluents A (water) and B (acetonitrile) contained 0.1% formic acid as ion-pairing modifier.

Data-dependent acquisition TripleTOF5600 mass spectrometer (Sciex, Concord, ON) was performed using following settings: 250 ms survey MS spectra (m/z 300–1500) was followed by up to 20 MS/MS measurements on the most intense parent ions (300 counts/s threshold, +2 to +4 charge state, m/z 100–1500 mass range for MS/MS, 100 ms each, high sensitivity mode). Previously targeted parent ions were excluded from repetitive MS/MS acquisition for 12 s (50 mDa mass tolerance).

### Data treatment and protein/peptide identification

Raw spectra files were converted into Mascot Generic File format (MGF) for peptide/protein identification by X!Tandem search algorithm [27]. Ten combined MGF files (each containing 21 MGFs of individual fractions) were created for subsequent protein identification and quantitation. The following X!Tandem search parameters were used: 20 and 50 ppm mass tolerance for parent and fragment ions, respectively; constant modification of Cys with iodoacetamide; default set post-translational modifications: oxidation of Met, Trp; N-terminal cyclization at Qln, Cys; N-terminal acetylation, phosphorylation (Ser, Thr, Tyr), deamidation (Asn and Gln); an expectation value cut-off of Log(e) < −1 for both proteins and peptides.

Spectra (in MGF format) and protein quantitation results are available at the University of California, San Diego’s MassIVE archive (massive.ucsd.edu) under the accession MSV000080890.

## Results

### Characterization of isolated MSC

**Fig. 1:**
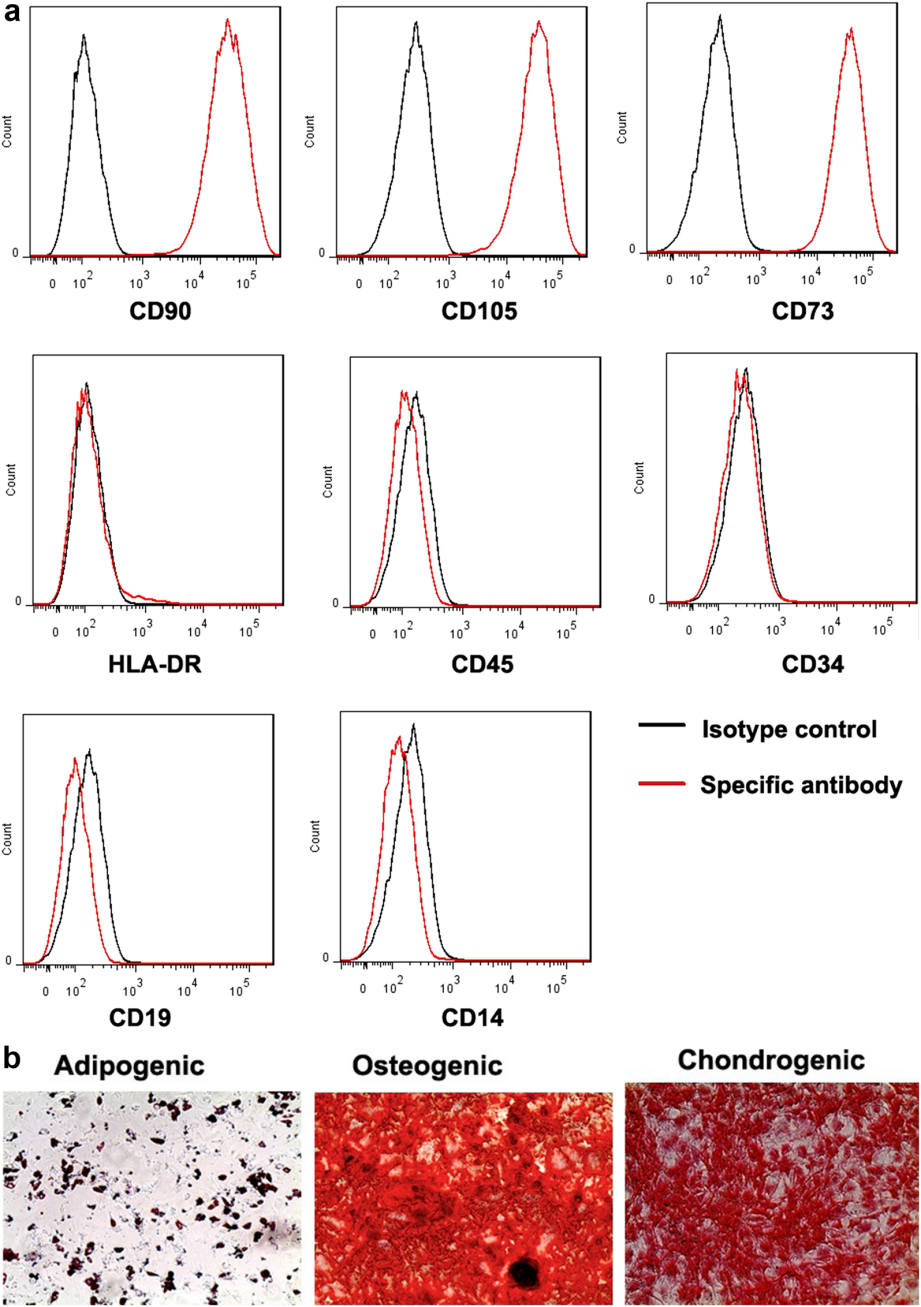
Characterization of MSC: representative data is provided for **a** flow cytometric analysis indicating that the cells displayed MSC markers CD90, CD105 and CD73 and the absence of lymphoid and myeloid cell markers and **b** differentiation capacity of MSC. Cells were assessed for their capacity to undergo Adipogenic (Oil Red O staining of lipid droplets after 14 days of induction); Osteogenic (Alizarin Red staining of calcium phosphate deposits produced by osteocytes after 21 days of induction; Chodrogenic (Safranin O staining of collagen matrix forming chondrocytes after 14 days of induction). (Original magnification ×100). All MSC met the International Society for Cell Therapy criteria for MSC

The isolated MSC were initially characterised to demonstrate that they expressed the appropriate surface markers and differentiation capacity as outlined by the International Society for Cell Therapy [23]. The cells expressed the MSC markers, CD90 (Thy-1), CD105 (endoglin) and CD 73 (ecto 5′nucleotidase). The cells lacked the pan-leukocyte marker CD45, the primitive hematopoietic and endothelial marker CD34, the B cell marker, CD19 and the macrophage/monocyte marker, CD14 as well as HLA-DR which is not expressed on MSC unless they have been activated (Fig. 1a). The capacity of the MSC to differentiate into chondrocytes, osteoblasts or adipocytes was also confirmed (Fig. 1b). Collectively these results indicate that the MSC isolates conformed to criteria for this designation.

### Protein identification and relative quantitation

Paired digests of cell lysates of untreated and IFN-γ treated MSC expanded from 5 separate healthy individuals were separated by 2D liquid chromatography and analysed by mass spectrometry. The resulting MGF spectra files for each analysis were sequentially concatenated and searched against a human subset of the Uniprot protein database (June 2015) using X!tandem (cyclone 2012.10.01.1). Confident protein identifications were selected based on the requirement for at least two distinct unique peptides each with expectation scores of −1.5 or lower and of −3 or lower for the protein. Peptide and protein identification statistics are summarized (Additional file 1: Table S1) which illustrate the significant and uniform depth of coverage across the five donor paired samples. A total of 8437 proteins were identified with ~4500 shared proteins being observed in all of the analyses.

Label-free protein expression values were computed as the sum of MS/MS fragment intensities for identified member peptides for each of the ten MS runs. In order to be included for quantification, at least two non-redundant peptide sequences were required for each protein. Protein level expression sums were mapped into a log_2_ scale and protein-by-protein differential analysis [D = (IFN intensity) − (CONTROL intensity)] was calculated across each of the donor cell pairs. These five donor protein-level differences each manifested as three Gaussian populations, with proteins observed in both modes forming the central population, and proteins observed uniquely in one mode or the other forming the other two populations. Selection of proteins for relative quantitation was based on a requirement that at least two non-redundant peptides were detected in the observed mode. Each central difference population was used for the normalization (mean = 0, SD = 1) of all three difference populations; while the resulting outer two difference population’s Z-scores were not directly informative of the probability of the difference being a random event. However, putting all measurements into a common scale made the comparative analysis across the five donors possible (Additional file 1: Table S1).

The data was analysed to identify those proteins in the IFN-γ treated population that showed a change of greater than two standard deviations from the mean of the overall protein change in the population (i.e. the mean difference of [D = (IFN intensity) − (CTL intensity)]. This corresponded to a ~3 fold difference in signal intensity because all five cell pairs had approximately the same difference distribution standard deviations. The criteria for assigning a protein differentially expressed status was based on a requirement that the protein displayed a consistent significant change in at least 3 of the 5 cell pairs following interferon treatment. This approach resulted in the identification of 169 proteins with increased expression and 41 proteins with decreased levels following interferon treatment (Tables 1A, B, 2A, B).

**Table 1:** Proteins that increased in MSC in response to IFN-γ treatment

ID	Gene	Description	Donor				
			4	69	74	140	150
(A)							
Q5T3U5	ABCC10	Multidrug resistance-associated protein 7	1.3	CTL	CTL	CTL	IFN
Q9UPQ3	AGAP1	Arf-GAP with GTPase, ANK repeat and PH domain-containing protein 1	4.3	6.3	CTL	4.0	0.8
Q9ULJ7	ANKRD50	Ankyrin repeat domain-containing protein 50	1.8	1.0	5.5	3.8	3.3
Q6ZW76	ANKS3	Ankyrin repeat and SAM domain-containing protein 3	CTL	CTL	CTL	0.6	CTL
Q8NCL9	APCDD1L	Protein APCDD1-like	CTL	2.4	CTL	1.9	3.0
Q9BSF8	BTBD10	BTB/PoZ domain-containing protein 10	CTL	CTL	CTL	CTL	CTL
Q8N5S9	CAMKK1	Calcium/calmodulin-dependent protein kinase kinase 1	CTL	CTL	0.8	3.9	CTL
P49674	CSNK1E	Casein kinase I isoform epsilon	4.2	6.9	1.4	CTL	5.3
Q9Y6M4	CSNK1G3	Casein kinase I isoform gamma-3	CTL	CTL	1.1	CTL	CTL
P39880	CUX1	Homeobox protein cut-like 1	1.1	CTL	CTL	13.7	CTL
Q9H8V3	ECT2	Protein ECT2	CTL	CTL	1.6	3.7	1.6
O95864	FADS2	Fatty acid desaturase 2	8.3	4.2	4.5	2.2	1.7
P02671	FGA	Fibrinogen alpha chain	CTL	CTL	73.9	0.5	4.4
P02679	FGG	Fibrinogen gamma chain	18.9	CTL	42.8	0.4	3.0
Q9NYZ3	GTSE1	G2 and S phase-expressed protein 1	CTL	CTL	CTL	CTL	4.0
Q9Y2K7	KDM2A	Lysine-specific demethylase 2A	5.4	0.3	1.1	CTL	6.4
Q9BVG8	KIFC3	Kinesin-like protein KIFC3	5.2	CTL	CTL	1.5	CTL
Q659C4	LARP1B	La-related protein 1B	3.5	CTL	CTL	CTL	CTL
Q15013	MAD2L1BP	MAD2L1-binding protein	CTL	CTL	CTL	0.7	CTL
Q07864	POLE	DNA polymerase epsilon catalytic subunit A	CTL	CTL	3.5	CTL	1.1
Q9P2K3	RCOR3	REST Corepressor 3	CTL	CTL	CTL	CTL	CTL
O15541	RNF113A	RING finger protein 113A	1.2	1.2	CTL	18.5	4.8
Q9GZN7	ROGDI	Protein rogdi homolog	CTL	0.0	CTL	CTL	1.8
Q99719	SEPT5	Septin-5	4.8	CTL	CTL	CTL	0.5
O95359	TACC2	Transforming acidic coiled-coil-containing protein 2	CTL	CTL	CTL	CTL	CTL
O15040	TECPR2	Tectonin beta-propeller repeat-containing protein 2	2.0	3.8	CTL	6.3	0.8
Q9P273	TENM3	Teneurin-3	3.9	CTL	3.2	2.1	1.3
Q86SZ2	TRAPPC6B	Trafficking protein particle complex subunit 6B	0.3	–	CTL	CTL	CTL
Q8IWR1	TRIM59	Tripartite motif-containing protein 59	CTL	3.2	4.0	CTL	0.9
Q9NPG3	UBN1	Ubinuclein-1	CTL	CTL	CTL	CTL	CTL
P62068	USP46	Ubiquitin carboxyl-terminal hydrolase 46	CTL	CTL	1.5	CTL	CTL
Q5ST30	VARS2	Valine–tRNA ligase, mitochondrial	CTL	CTL	1.9	3.5	CTL
Q9Y2K1	ZBTB1	Zinc finger and BTB domain-containing protein 1	0.5	CTL	CTL	7.3	CTL
Q9ULJ6	ZMIZ1	Zinc finger MIZ domain-containing protein 1	4.6	CTL	CTL	1.6	CTL
(B)							
Q9HCE6	ARHGEF10L	Rho guanine nucleotide exchange factor 10-like protein	IFN	–	IFN	IFN	–
Q6ICH7	ASPHD2	Aspartate beta-hydroxylase domain-containing protein 2	IFN	–	IFN	IFN	–
Q8WXX7	AUTS2	Autism susceptibility gene 2 protein	IFN	–	IFN	–	IFN
Q9BX70	BTBD2	BTB/POZ domain-containing protein 2	2.6	4.3	3.2	IFN	–
Q5VU97	CACHD1	VWFA and cache domain-containing protein 1	1.8	IFN	IFN	IFN	IFN
Q9BSQ5	CCM2	Cerebral cavernous malformations 2 protein	2.6	3.9	3.4	0.5	3.7
Q86YQ8	CPNE8	Copine-8	4.4	5.4	2.0	3.5	1.9
Q5H9U9	DDX60L	Probable ATP-dependentRNA helicase DDX60-like	IFN	IFN	IFN	8.1	8.2
Q96C10	DHX58	Probable ATP-dependentRNA helicase DHX58	IFN	IFN	IFN	IFN	IFN
Q96J88	EPSTI1	Epithelial-stromal interaction protein 1	IFN	IFN	IFN	IFN	IFN
Q9BTL3	FAM103A1	RNMT-activating mini protein	–	–	IFN	IFN	IFN
Q96MK3	FAM20A	Pseudokinase FAM20A	5.0	10.0	4.9	IFN	IFN
Q8IXL6	FAM20C	Extracellular serine/threonine protein kinase FAM20C	IFN	1.0	IFN	11.1	IFN
Q13480	GAB1	GRB2-associated-binding protein 1	IFN	2.2	4.8	IFN	–
Q14435	GALNT3	Polypeptide N-acetylgalactosaminyltransferase 3	IFN	12.1	8.5	3.6	IFN
P36269	GGT5	Gamma-glutamyltransferase 5	6.2	1.0	16.8	2.5	IFN
Q13547	HDAC1	Histone deacetylase 1	6.8	1.9	3.6	4.7	0.8
P01112	HRAS	GTPase HRas	13.6	1.4	1.2	4.1	IFN
P01579	IFNG	Interferon gamma	IFN	IFN	IFN	–	IFN
Q13572	ITPK1	Inositol-tetrakisphosphate 1-kinase	IFN	3.9	2.5	1.6	3.0
Q16363	LAMA4	Laminin subunit alpha-4	1.9	2.4	3.2	3.4	12.1
Q14392	LRRC32	Leucine-rich repeat-containing protein 32	IFN	IFN	IFN	–	IFN
P11137	MAP2	Microtubule-associated protein 2	–	–	IFN	IFN	IFN
Q96DP5	MTFMT	Methionyl-tRNA formyltransferase, mitochondrial	–	–	IFN	IFN	IFN
O75113	N4BP1	NEDD4-binding protein 1	3.5	2.0	2.1	4.6	3.5
Q7Z2Y5	NRK	Nik-related protein kinase	–	IFN	IFN	IFN	–
Q9Y5H3	PCDHGA10	Protocadherin gamma-A10	IFN	IFN	–	IFN	–
Q9H4M7	PLEKHA4	Pleckstrin homology domain-containing family A member 4	IFN	IFN	IFN	4.7	IFN
Q16647	PTGIS	Prostacyclin synthase	IFN	1.3	52.2	37.4	2.0
P35354	PTGS2	Prostaglandin G/H synthase 2	IFN	–	–	IFN	IFN
Q15262	PTPRK	Receptor-type tyrosine-protein phosphatase kappa	4.5	2.1	5.7	4.2	3.3
P05120	SERPINB2	Plasminogen activator inhibitor 2	IFN	IFN	IFN	1.0	CTL
P22732	SLC2A5	Solute carrier family 2, facilitated glucose transporter member 5	IFN	IFN	–	IFN	–
P84022	SMAD3	Mothers against decapentaplegic homolog 3	4.2	0.2	5.9	4.6	1.5
Q9BXI6	TBC1D1	TBC1 domain family member 1	3.8	IFN	10.0	1.5	2.6
Q9BXS4	TMEM59	Transmembrane protein 59	4.9	IFN	IFN	0.9	–
Q9BVA1	TUBB2B	Tubulin beta-2B chain	0.8	1.5	IFN	65.6	IFN
P22415	USF1	Upstream stimulatory factor 1	–	–	IFN	IFN	IFN
O75317	USP12	Ubiquitin carboxyl-terminal hydrolase 12	IFN	IFN	–	2.2	IFN
Q702N8	XIRP1	Xin actin-binding repeat-containing protein 1	IFN	IFN	IFN	IFN	7.3
Q9HCC9	ZFYVE28	Lateral signaling target protein 2 homolog	–	IFN	IFN	IFN	–

Proteins that were increased following interferon treatment with transcripts that are (A) designated as IFN-γ responsive or (B) not designated as IFN-γ responsive. Results are presented as fold increase following 20 h of IFN-γ treatment. IFN indicates only detected in interferon treated cells. CTL indicates only detected in untreated cells. – indicates not detected in indicated donor sample

**Table 2:** Proteins that decreased in MSC in response to IFN-γ treatment

ID	Gene	Description	Donor				
			4	69	74	140	150
(A)							
Q9H6X2	ANTXR1	Anthrax toxin receptor 1	1.1	3.9	CTL	2.2	CTL
Q8IZU8	DSEL	Dermatan-sulfate epimerase-like protein	CTL	–	CTL	–	CTL
P17342	NPR3	Atrial natriuretic peptide receptor 3	5.2	1.3	CTL	CTL	CTL
Q14123	PDE1C	Calcium/calmodulin-dependent 3′,5′-cyclic nucleotide phosphodiesterase 1C	1.1	6.2	CTL	CTL	2.1
O00411	POLRMT	DNA-directed RNA polymerase, mitochondrial	CTL	CTL	4.4	0.7	1.7
Q13885	TUBB2A	Tubulin beta-2A chain	–	–	6.5	11.6	6.4
Q86Y38	XYLT1	Xylosyltransferase 1	3.0	CTL	3.4	CTL	1.3
(B)							
Q9UPQ3	AGAP1	Arf-GAP with GTPase, ANK repeat and PH domain-containing protein 1	4.3	6.3	CTL	4.0	0.8
Q9ULJ7	ANKRD50	Ankyrin repeat domain-containing protein 50	1.8	1.0	5.5	3.8	3.3
Q6ZW76	ANKS3	Ankyrin repeat and SAM domain-containing protein 3	CTL	CTL	CTL	0.6	CTL
Q8NCL9	APCDD1L	Protein APCDD1-like	CTL	2.4	CTL	1.9	3.0
Q9BSF8	BTBD10	BTB/PoZ domain-containing protein 10	CTL	CTL	CTL	CTL	CTL
Q8N5S9	CAMKK1	Calcium/calmodulin-dependent protein kinase kinase 1	CTL	CTL	0.8	3.9	CTL
P49674	CSNK1E	Casein kinase I isoform epsilon	4.2	6.9	1.4	CTL	5.3
Q9Y6M4	CSNK1G3	Casein kinase I isoform gamma-3	CTL	CTL	1.1	CTL	CTL
P39880	CUX1	Homeobox protein cut-like 1	1.1	CTL	CTL	13.7	CTL
Q9H8V3	ECT2	Protein ECT2	CTL	CTL	1.6	3.7	1.6
O95864	FADS2	Fatty acid desaturase 2	8.3	4.2	4.5	2.2	1.7
P02671	FGA	Fibrinogen alpha chain	CTL	CTL	73.9	0.5	4.4
P02679	FGG	Fibrinogen gamma chain	18.9	CTL	42.8	0.4	3.0
Q9NYZ3	GTSE1	G2 and S phase-expressed protein 1	CTL	CTL	CTL	CTL	4.0
Q9Y2K7	KDM2A	Lysine-specific demethylase 2A	5.4	0.3	1.1	CTL	6.4
Q9BVG8	KIFC3	Kinesin-like protein KIFC3	5.2	CTL	CTL	1.5	CTL
Q659C4	LARP1B	La-related protein 1B	3.5	CTL	CTL	CTL	CTL
Q15013	MAD2L1BP	MAD2L1-binding protein	CTL	CTL	CTL	0.7	CTL
Q07864	POLE	DNA polymerase epsilon catalytic subunit A	CTL	CTL	3.5	CTL	1.1
Q9P2K3	RCOR3	REST Corepressor 3	CTL	CTL	CTL	CTL	CTL
O15541	RNF113A	RING finger protein 113A	1.2	1.2	CTL	18.5	4.8
Q9GZN7	ROGDI	Protein rogdi homolog	CTL	0.0	CTL	CTL	1.8
Q99719	SEPT5	Septin-5	4.8	CTL	CTL	CTL	0.5
O95359	TACC2	Transforming acidic coiled-coil-containing protein 2	CTL	CTL	CTL	CTL	CTL
O15040	TECPR2	Tectonin beta-propeller repeat-containing protein 2	2.0	3.8	CTL	6.3	0.8
Q9P273	TENM3	Teneurin-3	3.9	CTL	3.2	2.1	1.3
Q86SZ2	TRAPPC6B	Trafficking protein particle complex subunit 6B	0.3	–	CTL	CTL	CTL
Q8IWR1	TRIM59	Tripartite motif-containing protein 59	CTL	3.2	4.0	CTL	0.9
Q9NPG3	UBN1	Ubinuclein-1	CTL	CTL	CTL	CTL	CTL
P62068	USP46	Ubiquitin carboxyl-terminal hydrolase 46	CTL	CTL	1.5	CTL	CTL
Q5ST30	VARS2	Valine-tRNA ligase, mitochondrial	CTL	CTL	1.9	3.5	CTL
Q9Y2K1	ZBTB1	Zinc finger and BTB domain-containing protein 1	0.5	CTL	CTL	7.3	CTL
Q9ULJ6	ZMIZ1	Zinc finger MIZ domain-containing protein 1	4.6	CTL	CTL	1.6	CTL

Proteins that were decreased following interferon treatment with transcripts that are (A) designated as IFN-γ responsive or (B) not designated as IFN-γ responsive. Results are presented as fold decrease following 20 h of IFN-γ treatment. IFN indicates only detected in interferon treated cells. CTL indicates only detected in untreated cells. – indicates not detected in indicated donor sample

### Flow cytometric analysis of IFN-γ treated MSC

**Fig. 2:**
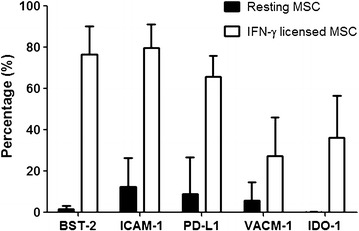
Induction of proteins by IFN-γ treatment. The percent of cells expressing the indicated proteins on untreated (resting) and IFN-γ treated MSC were determined by flow cytometry. The data represents the mean and SD of each analysis (n = 5 donors)

In an independent series of experiments the levels of expression of selected candidates (i.e. VCAM-1, ICAM1, IDO1, PDL1 and BST2) were compared between IFN-γ treated and the corresponding untreated MSC populations (Fig. 2). These proteins were all predicted from the proteomic analysis to increase following IFN-γ treatment. The flow cytometry results indicated that IDO1 and BST2 levels increased from being virtually undetectable to being highly expressed on all cells following treatment. The expression levels of PDL1, ICAM1 and VCAM1 increased from very low but to detectable levels to markedly increased expression in the IFN-γ treated group. These results were consistent with those of the mass spectrometry analysis and offered an independent validation of the proteomic expression results.

### Network and GO analysis

The list of significantly changed proteins identified in the proteomic analysis was submitted for STRING network analysis [28]. The analysis settings were: confidence level of 0.7 or greater using only evidence derived from databases and experiments. Analysis of the entire list indicated a highly organised and significant network (*p* = 0) with an average degree of 2.81 and a clustering coefficient of 9.45. There was a single dominant network consisting of 51 proteins which displayed a high level of connectivity. The remainder of the interacting proteins (n = 21) were associated with 7 smaller groups consisting of 2 or 3 interactors (Fig. 2). Thus of the 169 proteins that were increased following IFN-γ treatment ~40% (n = 68) were components of a protein–protein interaction network as determined by STRING. A separate analysis of the proteins that were decreased following IFN-γ treatment indicated that there were no significant protein–protein interactions in this group.

**Fig. 3:**
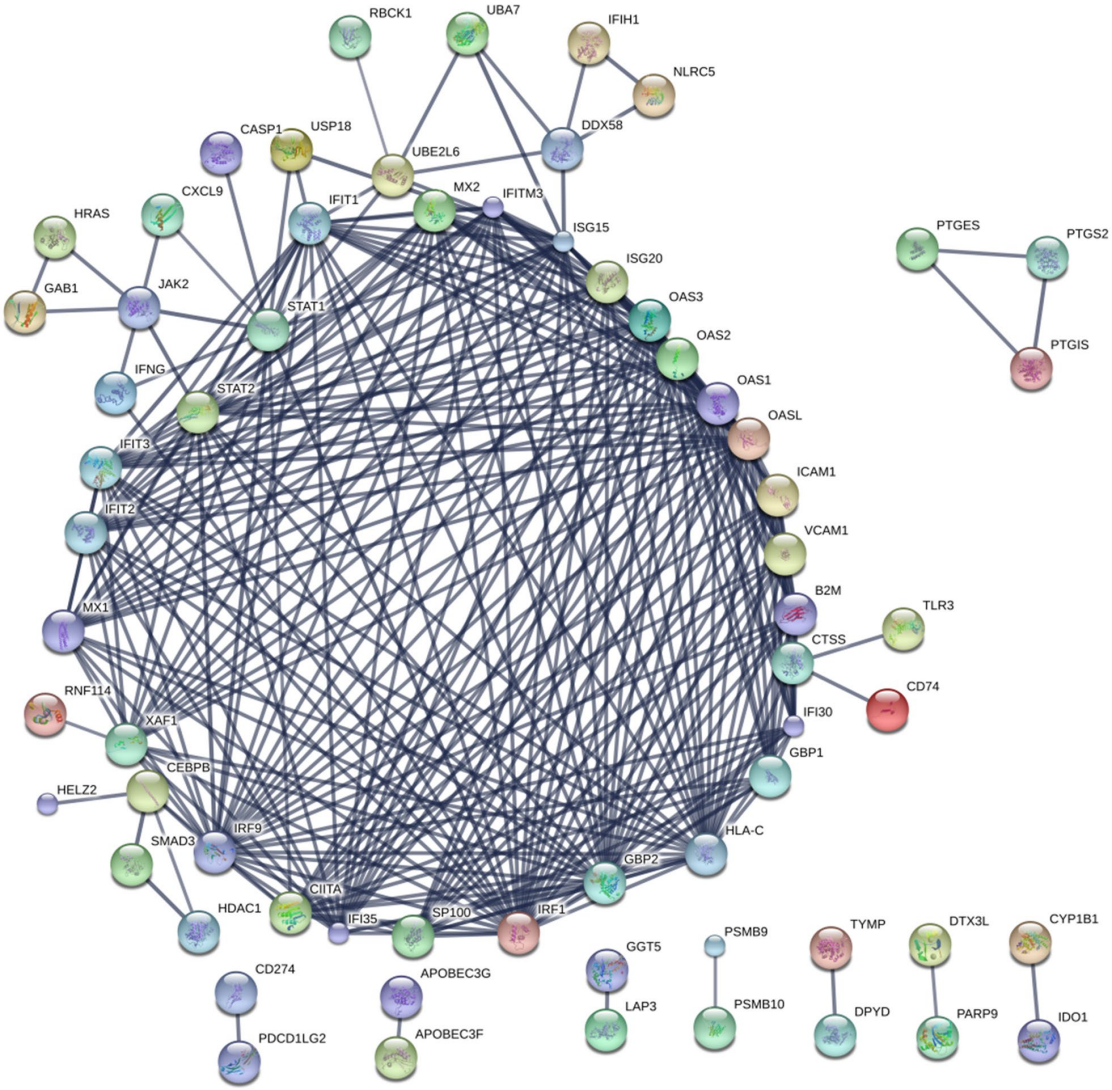
Network analysis proteins upregulated by IFN-γ treatment in MSC. The network analysis of the proteins significantly upregulated in human MSC following IFN-γ treatment. Results are based on STRING settings using experimental or database derived results. Note that only interacting proteins are included in this figure

Functional analysis of the altered proteins using GO indicated that there was a highly significant enrichment of proteins (n = 73) related to host defence responses (FDR 4.97 × 10^−33^). The proteins were enriched for “Interferon gamma-mediated signalling pathway” (n = 19, FDR 9.31 × 10^−21^). There was also a significant enrichment in proteins that were designated as involved in “Type I interferon signalling pathway” (n = 23, FDR 6.78 × 10^−27^). Although many of the upregulated proteins identified were common to both of the interferon signalling pathways, there were some clear differences in the proteins associated with the two pathways (Fig. 3). The remaining interacting proteins were components of shared pathways or processes (e.g. PTGES/PTGS2/PTGIS, APOBEC3F/APOBEC3G).

### Relationship between IFN-γ induced proteomic and transcriptomic changes

The protein lists were also submitted to the Interferome DB to determine if there was any evidence of corresponding altered gene expression levels in response to IFN-γ treatment [29]. To be consistent with our differential analysis selection criteria, the database was searched for absolute changes of at least twofold in IFN-γ treated samples relative to their untreated controls. The search was restricted to human studies and used only data derived from normal tissues or cells of any type. In total 135 of the 210 proteins that displayed changes in the protein levels in the IFN-γ treated cells were also reported in the Interferome DB (Tables 1A, 2A). However, approximately 35% of the proteins identified in the proteomic study have not been reported to be transcriptionally responsive to IFN-γ (Tables 1B, 2B).

It was noteworthy that a small subset of proteins upregulated in the interferon treated group and assigned to networks by the STRING analysis (HRAS, GAB1, HDAC1, SMAD3, GGT5, PTGS2, and PTGIS) have interactions with other IFN-γ regulated proteins. Each of these proteins was directly linked to a node in the network that was IFN-γ responsive.

### IFN-γ induced changes in membrane and secreted proteins

We were interested in the identification of IFN-γ induced membrane associated changes as these might represent useful biomarkers to measure the response of cells to IFN-γ treatment. The list of proteins displaying proteomic changes was analysed in Uniprot for expression of transmembrane regions [30]. A total of 47 proteins were predicted to be membrane proteins. The proteins are listed as those displaying either a gain or loss of expression in response to IFN-γ treatment (Table 3). The proteins were enriched for the KEGG pathways “Antigen processing and presentation” (CD74, HLA-C, HLA-DPA1, HLA-DRA, HLA-E, TAP1, TAPBP; FDR 1.07 × 10^−8^) and “Cell adhesion molecules” (CD274, HLA-C, HLA-DPA1, HLA-DRA, HLA-E, ICAM1, PDCD1LG2, VCAM1; FDR 2.3 × 10^−8^). Although not highly significant “Arachidonic acid metabolism” (GGT5, PTGES, PTGIS; FDR 0.00472) was also over represented in the membrane proteins. There were fewer membrane proteins (n = 8) identified that showed a decrease in expression following IFN-γ treatment. These proteins did not appear to be associated with any clearly defined process or functions.

**Table 3:** Membrane and secreted proteins with expression levels that were differentially influenced by IFN-γ treatment

Entry	Gene	TM	SP	RI^*^
Q9H6X2	ANTXR1	+	+	D
Q8NCL9	APCDD1L	+	+	D
O00478	BTN3A3	+	+	I
Q5VU97	CACHD1	+	+	I
Q9NZQ7	CD274	+	+	I
Q8IZU8	DSEL	+	+	D
P30504	HLA-C	+	+	I
P20036	HLA-DPA1	+	+	I
P01903	HLA-DRA	+	+	I
P13747	HLA-E	+	+	I
P30511	HLA-F	+	+	I
P05362	ICAM1	+	+	I
Q14392	LRRC32	+	+	I
P17342	NPR3	+	+	D
Q9Y5H3	PCDHGA10	+	+	I
Q9BQ51	PDCD1LG2	+	+	I
Q15262	PTPRK	+	+	I
Q8WVN6	SECTM1	+	+	I
O15533	TAPBP	+	+	I
Q9BX59	TAPBPL	+	+	I
O15455	TLR3	+	+	I
Q9BXS4	TMEM59	+	+	I
P19320	VCAM1	+	+	I
Q5T3U5	ABCC10	+		D
Q9ULC5	ACSL5	+		I
Q6ICH7	ASPHD2	+		I
O75110	ATP9A	+		I
O43286	B4GALT5	+		I
Q10589	BST2	+		I
P04233	CD74	+		I
O15247	CLIC2	+		I
O95864	FADS2	+		D
Q14435	GALNT3	+		I
P36269	GGT5	+		I
Q01628	IFITM3	+		I
O15162	PLSCR1	+		I
O14684	PTGES	+		I
Q16647	PTGIS	+		I
Q9Y666	SLC12A7	+		I
P22732	SLC2A5	+		I
Q03518	TAP1	+		I
Q03519	TAP2	+		I
Q9P273	TENM3	+		D
Q8IWR1	TRIM59	+		I
O95183	VAMP5	+		I
Q86Y07	VRK2	+		I
Q86Y38	XYLT1	+		D
O14791	APOL1		+	I
P61769	B2 M		+	I
P09871	C1S		+	I
P08603	CFH		+	I
P25774	CTSS		+	I
Q07325	CXCL9		+	I
Q96MK3	FAM20A		+	I
Q8IXL6	FAM20C		+	I
P02671	FGA		+	D
P02679	FGG		+	D
P13284	IFI30		+	I
P01579	IFNG		+	I
Q16363	LAMA4		+	I
Q08380	LGALS3BP		+	I
P35354	PTGS2		+	I
P05120	SERPINB2		+	I
P05155	SERPING1		+	I

*Proteins with transmembrane regions (TM) or signal peptides (SP) that were significantly changed in Response to IFN-γ treatment (RI) are indicated as either increased (I) or decreased (D) in the column labelled RI

**Fig. 4:**
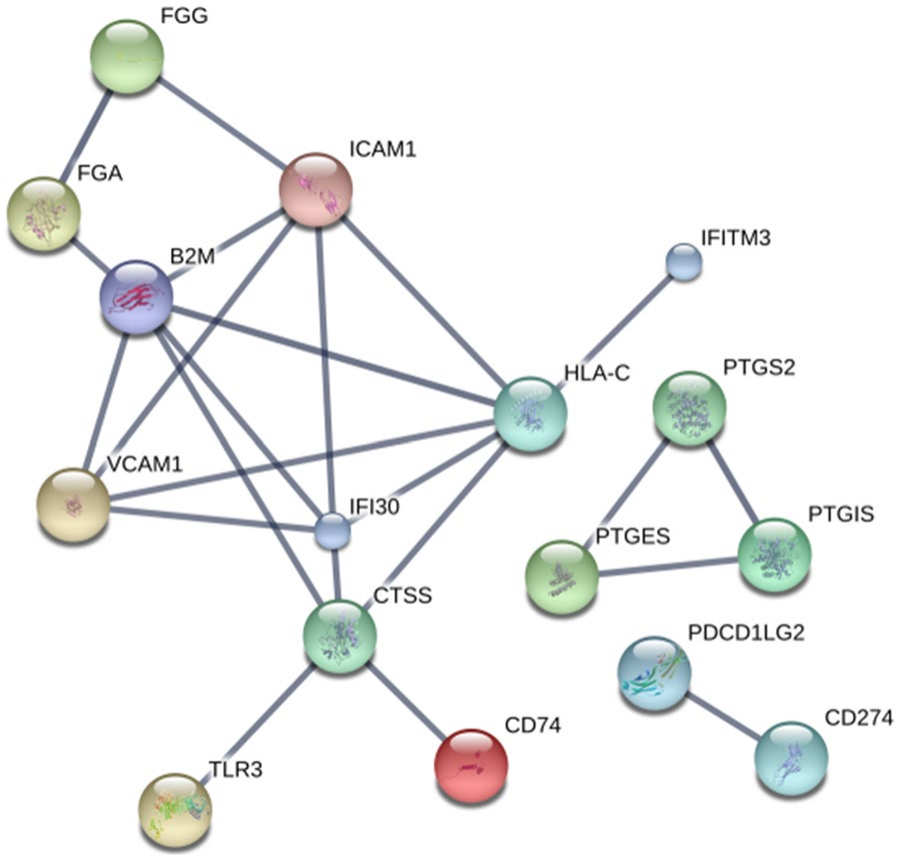
Network analysis of membrane and secreted proteins upregulated by IFN-γ treatment proteins in MSC. The network analysis of the membrane and secreted proteins significantly upregulated in human MSC following IFN-γ treatment. Results are based on STRING settings using experimental or database derived results. Note that only interacting proteins are included in this figure

There was also a subset of IFN-γ responsive proteins that were predicted to lack transmembrane sequences but to contain signal peptide sequences characteristic of secreted proteins (Table 3). STRING analysis of these proteins indicated protein interaction network associated with antigen processing and presentation and fibrinolysis. Notably fibrinogen which was downregulated following IFN-γ treatment was part of the network (Fig. 4).

## Discussion

The intent of this study was to identify IFN-γ induced protein changes in bone marrow derived MSC as this could provide new insights regarding the immunoregulatory capacities of these cells. Additionally protein compositional changes might also offer details of the differentiation associated biochemical processes involved in MSC licensing [26]. This made it feasible to perform a compositional comparison of cell pairs from different donors using label free quantitation [31]. The results indicated that only a small subset of 210 proteins changed significantly. Approximately 2/3 of these proteins displayed IFN-γ induced directionality changes in their expression levels which were consistent with those described for their transcripts in the literature or databases. The remainder of the proteins have not been reported to be IFN-γ responsive. These changes could be associated with either innate immune responsiveness or with the differentiation of the MSC following IFN-γ exposure. There is also the possibility that these proteins are actually IFN-γ responsive but their expression is post-translationally regulated. This could go undetected as the current classification of interferon responsiveness is largely based on changes in transcript levels following treatment, emphasizing the importance of proteomic analysis.

A significant proportion of the proteins that changed in response to IFN-γ were functionally enriched in biological processes involved in host defenses, especially those associated with antiviral responses. This was to be expected given the nature of the stimulus. Such responses included alterations in antigen processing, presentation and loading (B2M, TAPs1&2, TAPBP), foreign antigen and nucleic acid sensing (TLR3, OAS1/2/3), and ubiquitination/proteasome degradation pathways (TRIM 21, UBA7, USP18). There were also a number of other immunoregulatory proteins identified that have not previously been described in MSC cells (e.g. TAPBPL, THEMIS2, TNFAIP2).

Tapasin-related protein (TAPBPL) is a recently described regulator of peptide loading of Class I MHC complex [32–34]. This protein acts post TAP mediated loading of MHC to facilitate the exchange of high affinity peptides into the MHC. This provides a mechanism of assuring that MHC complexes remain charged with the peptide while expressed on the cell surface. This protein also provides an alternate mechanism of peptide loading of MHC I complexes, thus influencing the repertoire of peptides presented via class I molecules.

Thymocyte-selection-associated family member 2 (THEMIS2), also known as ICB1 (Induced on contact with basement membrane 1) increases the positive selection of murine B1 cells and germinal center B cells by self and foreign antigens [35]. This is achieved by lowering the threshold for B cell activation by low-avidity, but not high-avidity, antigens [36]. However, themis2 is not required for murine B cell development, activation, or antibody responses [37]. THEMIS2 has also been shown to function as a signalling scaffold in the murine macrophage line, RAW 264.7, displaying pathway-specific effects on TLR responses. The over-expression of themis2 enhanced the LPS-induced production of TNFα, but did not impact levels of IL-6 or Cox-2, nor TNF production induced by ligands for TLR2 or TLR3 [35].

Tumor necrosis factor alpha-induced protein 2 (TNFAIP2, also known as M-Sec) is A 73 kDa cytosolic protein that can also be induced by TNF α [38]. TNF α stimulation of Hela cells over or under expressing TNFAIP2/M-Sec leads respectively to reduced or enhanced levels of IL-8 in production. There was a direct correlation between poor outcomes in septic patients and single nucleotide polymorphisms in the TNFAIP2 promoter region which enhanced protein production [39]. These observations have led to the suggestion that TNFAIP2/M-Sec is an inhibitor of NF-κB activation. Functionally TNFAIP2/M-Sec is also essential for the formation of tunneling nanotubes (TNTs) [40]. TNTs are 50–200 nm diameter cellular protrusions that can connect cells up to of several cell diameters apart. These structures offer a mechanism for the transfer of small molecules as well as organelles and transport vesicles and they are of particular relevance to immune function. Dendritic cells and macrophages are among the few cell types that constitutively express TNFAIP2/M-Sec and they form TNT following antigen exposure. NK cells use TNT as an alternate mechanism to the immune synapse for transfer of lytic granules to their targets.

The previous examples represent IFN-γ responsive proteins that might contribute to the immunoregulatory activities of MSC. Indeed many are proposed mediators of MSC immunosuppressive activity (e.g. CD274, PDCD1LG2, ICAM1, IDO1, PTGES2) [2, 4]. However there is an additional group of proteins of potential immunological and immunoregulatory significance that are not currently described as IFN-γ responsive (e.g. PTGS2, PTGIS, FAM20A, FAM 20C and LRRC32/GARP).

Prostaglandin dependent mechanisms of MSC mediated immunosuppression have been described in a number of systems [41–43]. Thus it was of note that IFN-γ treated cells produced increased levels of prostaglandin G/H synthase 2 (PTGS2/COX-2), prostacyclin synthase (PTGIS) and prostaglandin E synthase (PTGES). This could provide a mechanism for increased production of prostaglandin H_2_ and downstream prostenoids. Although the inhibitory activity of PGE_2_ has been well documented prostacyclin has also been shown to have potent immunoregulatory properties. Prostacyclin synthase which generates prostacyclin is expressed by follicular dendritic cells in germinal centres of lymphoid organs [44]. PTGIS inhibits T cell activation while delaying the onset of apoptosis in these cells. Follicular dendritic-like HK cells have been shown to enhance the antigen presenting capacity of B cells, in part by induction of CD86 on the B cell surface. This effect is also observed with prostacyclin [3]. PTGIS contains an IFN-γ response element in 5′ region upstream of the translational initiation site [45]. However responsiveness to IFN-γ has not previously been reported.

The FAM20A and FAM20C are members of a family of secreted proteins [46]. FAM20A is differentially expressed in developing hematopoietic cells and it has recently been shown to be required for the appropriate compartmental distribution of and secretion of FAM20C [47]. FAM20C is an extracellular kinase that recognizes a consensus S-x-E/S sequence as a phosphorylation target. The broad specificity of the enzyme suggests that it may account for a large proportion of all extracellular phosphoproteins observed in humans [48]. Functionally the proteins targeted for phosphorylation are enriched in GO processes associated with adhesion, migration, invasion and wound healing. These observations have led to suggestions that FAM20C expression may contribute to the regulation of immune cell function and activity following IFN-γ treatment.

Epithelial stromal interaction protein 1 (EPSTI1) is a 37 kDa protein that was first identified in human breast cancer cells [49]. It has recently been shown to possess anti-apoptotic activity in human breast cancer cells [50]. The molecule is broadly expressed with highest message levels in the small intestine, spleen, salivary glands, and testes. EPSTI1 is an interferon response gene that is inducible by Type I interferon and the λ interferon (IL-28A) [51]. However, there have not been reports of IFN-γ responsiveness.

Leucine rich repeats containing 32/glycoprotein A repetitions predominant (LRRC32/GARP) is a type I membrane protein that binds and presents latent TGF-β in an active state on the surface of platelets, Tregs and MSC [52–54]. The protein has been demonstrated on the surface of human and murine MSC [55]. Our studies extend this by demonstrating that IFN-γ treatment enhanced production of LRRC32/GARP in human MSC. Although LRRC32/GARP message levels are only modestly upregulated by IFN-γ in other cell types, a recent publication demonstrated that protein levels were markedly increased murine macrophages following IFN-γ treatment.

The present studies demonstrate the value of direct measurements of protein expression patterns as many of the observed changes would apparently not have been predicted based on transcriptome analysis in other cell types. However, independent of these correlations it appears that there are a large number of immunoregulatory molecules that are altered following IFN-γ treatments and these may provide additional insights regarding the immunosuppressive mechanisms employed by licensed MSC. The identification of a significant number of membrane proteins that were differentially regulated following interferon treatment raises the possibility that these may represent potentially novel candidates for monitoring stem cell responsiveness to IFN-γ. While it remains to be determined which, if any, of these proteins actually correlates with the immunoregulatory activities of the IFN-γ treated MSC, the current results do indicate that are a large number of yet unassessed candidates. Additionally the present study offers one of the most in depth analysis of the basal and IFN-γ induced proteomes of bone marrow derived human MSC to date. This information may provide a baseline for the comparative analysis of the responses of these cells to other licensing agents.

## Additional file

**Additional file 1: Table S1.** Summary data of identifications, intensities and normalised z scores of differences (i.e. of [D = (IFN intensity)-(CTL intensity)]. CTL and IFN refer respectively to cumulative peak intensities of peptides (log 2) for a given protein in untreated and interferon treated pairs of cells from the same donor. Z corresponds to the normalised z score for the differences observed for each protein (i.e. of [D = (IFN intensity)-(CTL intensity)] relative to the mean of the distribution within their corresponding donor pair.
